# Anaphylactic shock following intraurethral lidocaine administration during transurethral resection of the prostate

**DOI:** 10.4103/0970-1591.38616

**Published:** 2008

**Authors:** M. Sinha, R. Sinha

**Affiliations:** Department of Surgery, Tata Main Hospital, Jamshedpur, Jharkhand, India; 1 Department of Anesthesia, Tata Main Hospital, Jamshedpur, Jharkhand, India

**Keywords:** Anaphylactic shock, intraurethral, lidocaine

## Abstract

Anaphylactic shock was noted following an apparently uneventful transurethral resection of the prostate (TURP). Lidocaine jelly was used prior to urethral dilatation and before placement of three-way Foley. Lidocaine sensitivity was diagnosed serendipitously when lidocaine jelly was used for application of ECG electrodes. Anaphylaxis may be one of the rare differentials to be considered in a patient with postoperative shock following TURP. This report highlights a potentially fatal complication of an apparently innocuous and ubiquitous urological use of lidocaine.

## CASE REPORT

A 65-year-old gentleman underwent an apparently uneventful transurethral resection of the prostate (TURP). Spinal anesthesia was given using 0.5% bupivacaine. Amikacin was administered before surgery. The 40-minutes TURP was apparently uneventful. No other intravenous drugs were administered. Intraurethral lidocaine jelly consisting of lidocaine hydrochloride 2%, methyl paraben 0.061%, propyl paraben 0.027% was administered at the beginning of the procedure prior to urethral dilatation and again towards the end of the procedure before placement of the Foley catheter. Ten minutes after completion of TURP the patient developed a severe urticarial rash all over the body. Blood pressure was 70/50 mmHg. There was no fever, bronchospasm or laryngospasm. A diagnosis of anaphylactic shock was made. One hundred mg hydrocortisone, 50 mg phenylephrine, 25 mg pheniramine maleate and crystalloids were administered. Blood pressure rose to normal levels and the rash disappeared. A list of possible causative agents used during surgery was evaluated. All intravenous fluid and glycine bottles were rechecked for contaminants. Initially the offending agent could not be identified. As the patient had a hypotensive episode an ECG was asked for. Serendipitously the nurse applied lidocaine jelly at the electrode sites. The patient developed localized wheal and flare reactions at these sites. This confirmed lidocaine sensitivity. Keeping the chronology of events in mind we deduce that the initial administration of lidocaine remained intravesical and got washed out. The jelly administered prior to Foley catheter placement would have got absorbed from the raw prostatic fossa and resulted in anaphylactic shock. Retrospectively the patient was asked for any relevant history. Following an earlier cataract surgery patient had some swelling around the eyes. The patient had been told that it was an infection but it could have been a manifestation of lidocaine sensitivity.

## DISCUSSION

The estimated incidence of anaphylaxis is one in 6500 administrations of neuromuscular blocking agents.[1] The usual allergens responsible in lidocaine gels are the parabens preservatives and chlorhexidine. The lidocaine component is not usually involved. The preparation used in this case did not contain chlorhexidine and we believe that the parabens component was the responsible allergen. As most preparations used in urological surgery do contain parabens preservatives further component testing for sensitivity was not considered necessary. Moreover the patient declined consent for the same. While anaphylaxis can occur due to any drug the notable feature of this case is the intraurethral route of lidocaine administration. Anaphylaxis may be one of the rare differentials to be considered in a patient with postoperative shock following TURP. Although Carr has reported anaphylaxis following intraurethral lidocaine jelly and flexible cystoscopy we could not find any similar cases reported during TURP.[2] We did find convulsions reported following intravascular absorption of lidocaine during intraurethral instillation.[3] This case serves as a reminder of a possible fatal complication of an extremely common urological use of lidocaine.
